# Clinical utility of the Revised International Staging System in unselected patients with newly diagnosed and relapsed multiple myeloma

**DOI:** 10.1038/bcj.2017.13

**Published:** 2017-02-17

**Authors:** N Tandon, S V Rajkumar, B LaPlant, A Pettinger, M Q Lacy, A Dispenzieri, F K Buadi, M A Gertz, S R Hayman, N Leung, R S Go, D Dingli, P Kapoor, Y Lin, Y L Hwa, A L Fonder, M A Hobbs, S R Zeldenrust, J A Lust, W I Gonsalves, S J Russell, S K Kumar

**Affiliations:** 1 Division of Hematology, Mayo Clinic, Rochester, MN, USA

## Abstract

We analyzed the utility of Revised International staging system (RISS) in an unselected cohort of newly diagnosed multiple myeloma (NDMM; cohort 1), and relapsed/refractory multiple myeloma (RRMM; cohort 2) patients. Cohort 1 included 1900 patients seen within 90 days of diagnosis, from 2005 to 2015, while cohort 2 had 887 patients enrolled in 23 clinical trials at Mayo Clinic. The overall survival (OS) and progression-free survival (PFS) was calculated from the time since diagnosis or trial registration. The median estimated follow up was 5 and 2.3 years for Cohorts 1 and 2, respectively. Among 1067 patients evaluable in Cohort 1, the median OS and PFS was 10 and 2.8 years for RISS stage I, 6 and 2.7 years for RISS stage II and 2.6 and 1.3 years for RISS stage III (*P*<0.0001). Among 456 patients evaluable in Cohort 2, the median OS and PFS was 4.3 and 1.1 years for RISS stage I, 2 and 0.5 years for RISS stage II and 0.8 and 0.2 years for RISS stage III (*P*<0.0001). In conclusions, RISS gives a better differentiation of NDMM as well as RRMM patients into three survival subgroups and should be used to stratify patients in future clinical trials.

## Introduction

Multiple myeloma (MM) is a very heterogeneous disease with nearly a quarter of patients dying within 3 years of diagnosis and others maintaining durable disease control for more than 10 years.^1, 2^ For this reason, various prognostic factors and staging systems have been developed to predict the disease outcomes. The Durie/Salmon staging sytem used hemoglobin, calcium level, the type and level of monoclonal protein and number of bone lesions to predict myeloma cell tumor burden and long-term outcomes.^3^ Subsequently, the International Staging System (ISS) was developed, which identified three patient groups with different survival based on serum β2- microglobulin (β2m) and serum albumin. High serum β2m reflected high tumor burden and reduced renal function, while low serum albumin is thought to be mediated by the effects of inflammatory cytokines like interleukin-6 (IL-6).^4^

However, ISS did not incorporate one of the most important prognostic factors in MM, namely chromosomal abnormalities (CA). In patients with newly diagnosed MM (NDMM), high-risk disease is characterized by the presence of del (17p), t(4; 14) (p16;q32) or t(14;16)(q32;q23) detected by interphase fluorescent *in situ* hybridization.^5, 6^ High serum lactate dehydrogenase (LDH) has been linked to shorter OS in MM and likely reflects disease aggressiveness and drug resistance, and may also be an indicator of extramedullary disease.^7, 8^ The revised ISS stage (Revised International staging system (RISS)) was developed by pooling data from 4445 patients with NDMM enrolled on 11 international trials. It combines the ISS with high-risk CA [del (17p), t(4; 14) (p16; q32) or t(14; 16) (q32; q23)] and serum LDH to classify patients into three risk groups. The 5-year overall survival (OS) of patients with stage I, II and III RISS was 82, 62 and 40, while the 5-year progression-free survival (PFS) was 55%, 36% and 24%, respectively.^9^

Unlike ISS, RISS has been derived from data obtained only from patients enrolled in clinical trials. Clinical trials almost always exclude patients with serious comorbidities and poor performance making it less applicable to real-life scenario.^10, 11^ Finally, RISS development only used data from previously untreated symptomatic MM, and its utility in relapsed disease is less clear.^4, 9^ Hence, we examined the utility of RISS in an unselected cohort of patients with NDMM as well as in a selected group of patients with relapsed/refractory MM (RRMM).

## Patients and methods

### Patients

The purpose of our study was to analyze the clinical utility of the RISS in patients with NDMM in a non-clinical trial setting and in patients with RRMM. To address these issues, we studied two cohorts of patients. The first cohort (NDMM cohort; cohort 1) included 1900 consecutive patients with NDMM seen at Mayo Clinic within 90 days of diagnosis, between January 2005 and December 2015. Their clinical and laboratory data were collected by chart review and analyzed retrospectively. The second cohort (RRMM cohort; cohort 2) included 887 patients with RRMM enrolled in different clinical trials at Mayo Clinic.

### ISS and RISS

ISS stage I is defined as serum β2m <3.5 mg/l and serum albumin level ⩾3.5 g/dl, stage III is defined as serum β2m>5.5 mg/l and stage II includes all remaining patients.^4^ The RISS stage I includes patients with ISS stage I with no high-risk CA [del(17p) and/or t(4;14) and/or t(14;16)], and normal LDH level (less than the upper limit of normal range), stage III includes patients with ISS stage III and either high-risk CA or high LDH level, and stage II includes all the other possible combinations.^9^ The value of all four variables (β2m, albumin, CA and LDH) was available for 1067 patients in the NDMM cohort, though RISS could be calculated in 1352 patients on the basis on the availability of either CA or LDH. RISS staging was analyzed in 456 assessable cases in the RRMM cohort with complete data.

### Prognostic risk score

We also examined a simpler approach that utilized equal weight for each of the poor prognostic factors. The variables used to calculate the RISS stage were assigned a value of either 0, if favorable, or 1, if unfavorable as follows: β2m (<5.5 mg/l versus ⩾5.5 mg/l), albumin (⩾3.5 g/dl versus <3.5 g/dl), LDH (⩽upper limit of normal range versus >upper limit of normal range), high-risk translocation (absence versus presence) and deletion 17p (absence versus presence). The values of these four variables were then added together to arrive at a final score.

### Statistical analysis

The primary end point was OS, defined as the time from diagnosis (NDMM cohort) or trial registration (RRMM cohort) until death from any cause or until the patient was last known to be alive. The secondary end point of PFS was defined as the time from diagnosis or trial registration until progression or death as a result of any cause or until the last date the patient was known to be progression free, whichever occurred first. The OS and PFS curves were estimated using the Kaplan–Meier method and their differences were analyzed using the two-sided log-rank test. All reported *P*-values were two sided at the conventional 5% significance level. Data were analyzed with SAS version 9.4 software (SAS Institute Inc., Cary, NC, USA).

## Results

### Baseline characteristics and treatments

In the NDMM cohort, the median age was 65.0 years (range, 22–95 years) and 59.9% were male. Among these, 1751 patients (92.2%) received novel drugs (immunomodulatory drugs (IMIDs) or proteasome inhibitors (PIs)) as induction or initial therapy with or without autologous stem cell transplant and maintenance treatment. Overall, RISS could be calculated in 71.2% (*n*=1352) of patients; among which 229 patients (17.0%) were RISS stage I, 938 patients (69.4%) were RISS stage II and 185 patients (13.7%) were RISS stage III.

In RRMM cohort, the median age was 65.0 years (range, 32–90 years) and 59.6% were males. Six-hundred fifty-four patients (73.7%) received novel drugs as part of trial therapy. Overall, RISS could be calculated in 51.4% (*n*=456) of patients; among which 104 patients (22.8%) were RISS stage I, 294 patients (64.5%) were RISS stage II and 58 patients (12.7%) were RISS stage III. The baseline characteristics of both the cohorts are listed in Table 1.

### RISS staging and prognostication

**Figure 1:**
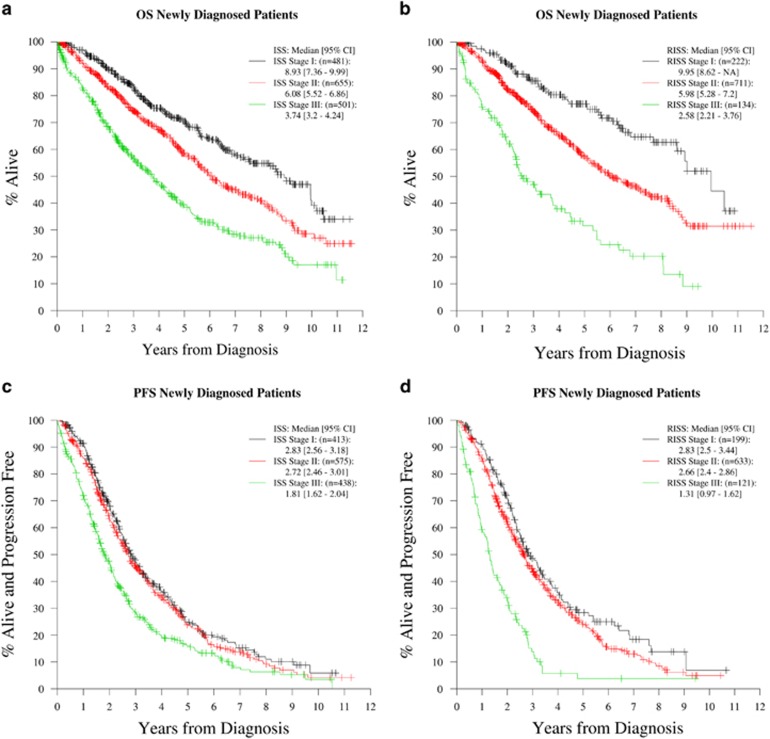
OS and PFS curves for different ISS and RISS stages among patients with NDMM (Cohort 1). (**a**) OS curves for all ISS stages in patients with NDMM. (**b**) OS curves for all RISS stages in patients with NDMM. (**c**) PFS curves for all ISS stages in patients with NDMM. (**d**) PFS curves for all RISS stages in patients with NDMM.

**Figure 2:**
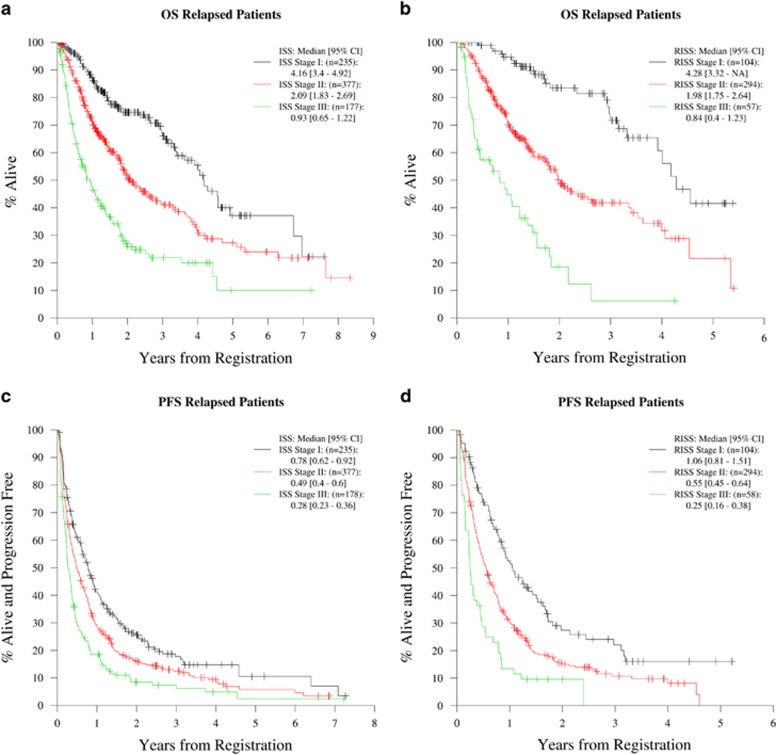
OS and PFS curves for different ISS and RISS stages among patients with RRMM (Cohort 2). (**a**) OS curves for all ISS stages in patients with RRMM. (**b**) OS curves for all RISS stages in patients with RRMM. (**c**) PFS curves for all ISS stages in patients with RRMM. (**d**) PFS curves for all RISS stages in patients with RRMM.

The median estimated follow up was 5 years (95% confidence interval (CI); 4.8, 5.5 years) for NDMM cohort and 2.3 years (interquartile range; 2.1, 2.6 years) for the RRMM cohort. The main analysis included 1067 assessable cases in the NDMM cohort and 456 assessable cases in the RRMM cohort with RISS calculated on the basis of complete data on all four variables. The median and 5-year estimates of OS and PFS for the NDMM cohort based on ISS (Figures 1a and c) and RISS (Figures 1b and d) stages are depicted in Table 2. The outcomes according to RISS staging done on a total of 1352 patients in whom RISS could be calculated on the basis of availability of either CA or LDH is depicted in the Supplementary Table 1. The median and 2-year estimates of OS and PFS for the RRMM cohort according to the ISS (Figures 2a and c) and RISS staging (Figures 2b and d) are listed in Table 3.

We also looked at the differences in outcomes in patients belonging to various RISS stages among patients diagnosed between 2005 and 2010 as compared with those diagnosed between 2011 and 2015. The median OS was 9.8 years (95% CI; 8.6 years, not reached) versus not reached for patients belonging to RISS stage I (*P*=0.45), 5.9 years (95% CI; 5.2, 6.7 years) versus 5.4 years (95% CI; 5.4 years, not reached) for patients belonging to RISS stage II (*P*=0.13), and 2.7 years (95% CI; 2.2, 3.6 years) versus 3.2 years (95% CI; 1.9 years, not reached) for patients belonging to RISS stage III (*P*=0.6). The median PFS among patients diagnosed from 2005 to 2010 versus those diagnosed from 2011 to 2015 was 2.8 years (95% CI; 2.5, 3.6 years) versus 3.2 years (95% CI; 2.3, 3.4 years) for patients belonging to RISS stage I (*P*=0.56), 2.3 years (95% CI; 2.1, 2.6 years) versus 2.7 years (95% CI; 2.5, 3 years) for patients belonging to RISS stage II (*P*=0.45), and 1.3 years (95% CI; 0.9, 1.6 years) versus 1.4 years (95% CI; 1.1, 2.1 years) for patients belonging to RISS stage III (*P*=0.55). Hence, although the median OS and PFS has improved in 2011–2015 as compared with 2005–2010 among all RISS stages; however, the difference is not statistically significant.

### Prognostic risk score

**Figure 3:**
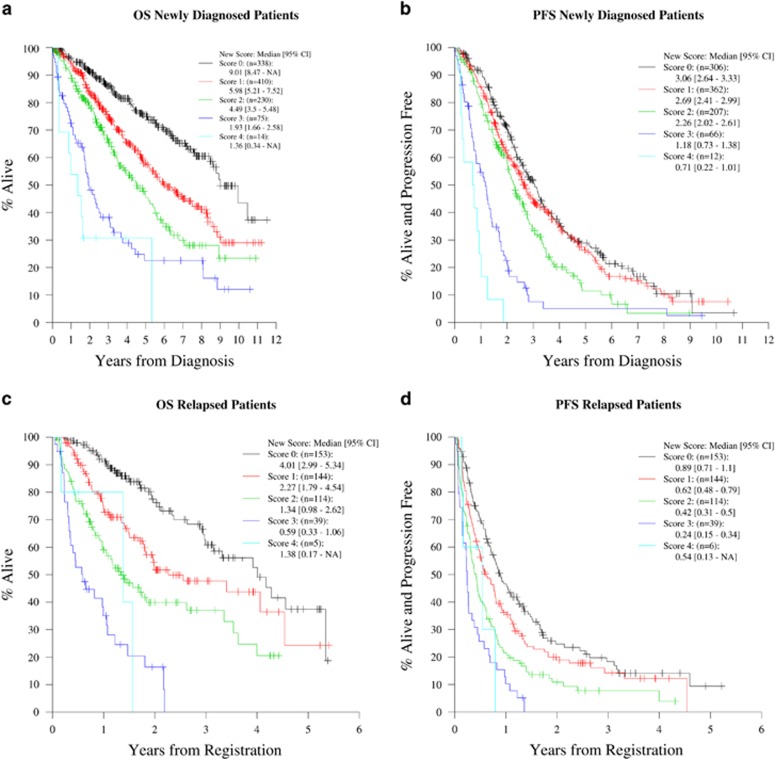
OS and PFS curves for different prognostic risk scores among patients with NDMM (Cohort 1) and RRMM (Cohort2). (**a**) OS curves for all risk scores in patients with NDMM. (**b**) PFS curves for all risk scores in patients with NDMM. (**c**) OS curves for all risk scores in patients with RRMM. (**d**) PFS curves for all risk scores in patients with RRMM.

The variables used to determine the RISS stage were used to calculate a prognostic risk score in both the cohorts as described in Methods. Among NDMM, there were 1067 assessable patients for whom the complete data for all the four variables were available. Among these patients, 31.7% (*n*=338), 38.4% (*n*=410), 21.6% (*n*=230), 7.0% (*n*=75) and 1.3% (*n*=14) had risk scores of 0, 1, 2, 3 and 4 respectively. The median and 5-year OS (Figure 3a) and PFS (Figure 3b) estimates for this cohort are shown in Table 2.

In the RRMM cohort, there were 456 assessable patients for whom the complete data for all the four variables were available. Among these patients, 33.6% (*n*=153) had a final risk score of 0, 31.6% (*n*=144) had a score of 1, 25.0% (*n*=114) had a score of 2, 8.6% (*n*=39) had a score of 3 and only 1.3% (*n*=6) had a final risk score of 4. The median and 2-year OS (Figure 3c) and PFS (Figure 3d) estimates for this cohort are listed in Table 3.

## Discussion

In this study, we depicted that the RISS staging system is a simple and easily applicable prognostic model, which gives a better distinction of patients with NDMM as well as RRMM into three survival groups. Patients with NDMM (outside clinical trials) with RISS stage I, II and III had a 5-year OS rate of 76.3%, 55.7% and 29.5%, respectively, while patients with RRMM with RISS stage I, II and III had a 2-year OS rate of 83.5, 48.7 and 18.5. We also demonstrated that as compared with patients with NDMM diagnosed between 2005 and 2010, those diagnosed between 2011 and 2015, have better OS and PFS among all RISS groups, though the results are not statistically significant. In addition, we also showed that a simpler approach giving equal weight to each of the four prognostic variables can also be used to stratify patients with NDMM and RRMM.

Although ISS staging system is a powerful prognostic tool reflecting the patient's status and the tumor burden, it does not account for the biological factors which have a major role in disease evolution and resistance to treatment. The CA not only depict the prognosis of patients with myeloma, but also affect clinical presentation and management strategies.^12^ Also, high serum LDH has a major impact on the survival of myeloma patients even when they belong to a low or intermediate ISS subgroup.^13^ Hence, the incorporation of CA and LDH levels into ISS to formulate RISS staging system is highly relevant.

In addition, it should be noted that serum β2m levels may be elevated in several benign conditions such as chronic inflammation, liver disease, renal dysfunction and some acute viral infections, apart from being prognostic in lymphoproliferative malignancies, especially MM.^14^ Hence, although the criteria for ISS stage I is very specific, any patient with elevated serum β2m falls in ISS stage III. Based on the RISS staging system, 13.7% of NDMM patients belong to the poor prognostic RISS stage III. Interestingly, Shaughnessy *et al.* identified a 70-gene subset as a predictor of outcome after studying the gene expression profile of 532 patients with NDMM and found that the high-risk score was present in 13% of patients with shorter durations of complete remission, event-free survival (EFS), and OS.^15^

The International Myeloma Working Group (IMWG) report on RISS analyzed a large sample size of 4445 patients with NDMM enrolled onto 11 international clinical trials. The Surveillance, Epidemiology and End Results analysis from 2008 to 2012 states that MM is most frequently diagnosed among people aged 65–74 years with 61.8% of patients aged ⩾65 years.^16^ This elderly population is not well represented in the IMWG report with only 35% of patients were older than 65 years. In our study, 46.89% of patients with NDMM in Cohort 1 were aged more than 65 years. In addition, all patients analyzed in IMWG study received novel agents with or without autologous stem cell transplant as part of their upfront treatment. Our study, however, has included all patients seen at Mayo Clinic over a span of 10 years from 2005 onwards. Hence, 149 patients (7.84%) did not receive novel agents or autologous stem cell transplant for their initial treatment. As in the report by IMWG, the majority of patients (69.33% in our study versus 62% in IMWG study), belonged to intermediate risk category. This distribution aids in better survival differentiation among the 3 RISS stages.

Randomized clinical trials represent a final step in evaluating the efficacy of any new treatment. However, <3% of adult cancer patients participate in clinical trials in the United States.^17^ Trials may not be available for patients willing to participate, or when they are available, patients are not enrolled because they do not meet trial eligibility criteria.^18, 19^ Usually, patients are excluded on basis of criteria pertaining to age, comorbidities and performance status.^20^ Various studies have demonstrated positive evidence that participation in trials improves outcomes.^20, 21, 22^ Even after matching patients for age, stage, *de novo* presentation, and treatment, trial patients could still benefit from changes in behavior or outlook associated with being under observation (the ‘Hawthorne' effect) or from care that is administered according to strict protocol.^23, 24, 25^ Therefore, it was very important to assess the clinical utility of the RISS staging system in real-life patients outside clinical trials.

MM remains an incurable disease and nearly all patients with the disease relapse and eventually succumb to refractory disease. The extent of disease at relapse, the type and response to previous therapy as well as the time of relapse affect prognosis of such patients. Kumar *et al.* analyzed the outcomes of 286 patients with relapsed MM, who were refractory to bortezomib and were relapsed following, refractory to or ineligible to receive, an IMiD based on ISS stage at time of enrollment (T0) in the study. He showed that the ISS stage was prognostic for OS following T0 with median survivals of 12, 8 and 4 months for ISS stages 1, 2 and 3, respectively.^26^ However, the RISS staging system was yet to be validated in patients with RRMM.

In addition, we demonstrated that as compared with patients with NDMM diagnosed between 2005 and 2010, the median OS and PFS in those diagnosed between 2011 and 2015, have improved for RISS stage I, II as well as III, although the results are not statistically significant. Previously, a retrospective analysis of 1038 patients also showed that the median OS increased from 4.6 years to 6.1 years for 2001–2005 cohort versus 2006–2010 cohort (*P*=0.02). The improvement in outcomes was linked to use of one or more novel agents like bortezomib, lenalidomide and thalidomide.^1^ In the United States, four drugs (panobinostat, ixazomib, aratumumab and elotuzumab) were approved for the treatment of MM in 2015.^27^ Hence, we expect that the impact of incorporation of these agents will be seen over time.

The retrospective nature and single institutional experience of this study does limit the scope of its conclusions. However, after reviewing the literature, we concluded that this is one of the largest known series reported on these patient populations. Our study not only validated the RISS in patients with NDMM as well as RRMM but also explored a new prognostic risk score using all the four prognostic variables.

## Conclusion

In conclusion, MM is a heterogeneous disease and requires a simple, reliable and easily applicable staging system that combines clinical and biological disease related prognostic factors and can be applied for universal patient classification. The RISS combines the prognostic power of high-risk CA and LDH with ISS score and gives a better differentiation of MM patients into three survival subgroups. In this study, we showed that the difference in survival outcomes retain statistical significance in an unselected cohort of patients with NDMM as well as in a group of patients with RRMM enrolled in various clinical trials. Hence, it should be used to stratify patients in future clinical trials to guide towards more effective personalized treatment.

We also found that though the median OS and PFS has improved in 2011–2015 as compared with 2005–2010 among all RISS stages; however, the difference is not statistically significant. In addition, we analyzed a simpler approach giving equal weight to each of the four prognostic variables, which can also be used to stratify patients with NDMM and RRMM. Gene expression profiling and assessment of minimal residual disease by multiparametric flow cytometry or molecular methods are currently being studied for detailed analysis and prognostication of MM and may be incorporated in future staging algorithms.

## Figures and Tables

**Table 1:** Baseline characteristics for Cohorts 1 and 2

*Characteristic*	*Number of patients (%)*	
	*Cohort 1 (*N=*1900)*	*Cohort 2 (*N=*887)*
*Age, years*		
⩽65	1009 (53.1%)	465 (52.4%)
>65	891 (46.9%)	422 (47.6%)
		
*Sex*		
Male	1137 (59.9%)	529 (59.6%)
Female	763 (40.2%)	358 (40.4%)
		
*ISS stage*		
I	481 (25.0%)	235 (26.5%)
II	653 (34.4%)	377 (42.5%)
III	501 (26.4%)	178 (20.1%)
Missing	265 (14.0%)	97 (10.9%)
		
*High-risk CA by iFISH*		
Absent	1071 (56.4%)	346 (39.0%)
Present	297 (16.0%)	126 (14.2%)
Missing	532 (28.0%)	415 (46.8%)
		
*LDH level*		
Normal	1258 (66.2%)	673 (75.9%)
Elevated	268 (14.1%)	182 (20.5%)
Missing	374 (19.7%)	32 (3.6%)
		
*Treatment*		
IMIDs	1304 (68.6%)	458 (51.6%)
PIs	877 (46.2%)	196 (22.1%)
ASCT	247 (13%)	0 (0%)
Other	0 (0%)	233 (26.3%)
Missing	9 (0.47%)	0 (0%)

Abbreviations: ASCT, autologous stem cell transplant; CA, chromosomal abnormalities; iFISH, fluorescent *in situ* hybridization; IMID, immunomodulatory drugs; ISS, International Staging System; LDH, lactate dehydrogenase; PIs, proteasome inhibitors.

**Table 2:** OS and PFS analysis across three different diagnostic scores in newly diagnosed patients (Cohort 1)

*Diagnostic test*	*Cohort*	*Median OS years (95% CI)*	*5-Year OS % (95% CI)*	*Median PFS years (95% CI)*	*5-Year PFS % (95% CI)*
ISS	ISS-I	8.9 (7.36–9.99)	70.4 (65.2–75.1)	2.83 (2.56–3.18)	25.3 (20.2–30.7)
	ISS-II	6.08 (5.52–6.86)	58.5 (53.8–62.9)	2.72 (2.46–2.98)	23.7 (19.6–27.9)
	ISS-III	3.74 (3.20–4.24)	38.5 (33.3–43.6)	1.81 (1.62–2.04)	16.0 (12.2–20.2)
	Total	5.74 (5.48–6.36)	55.7 (52.8–58.5%)	2.46 (2.32–2.61)	21.8 (19.2–24.4)
RISS	RISS-I	9.95 (8.62–NA)	77 (69.4–82.9)	2.83 (2.5–3.44)	28.4 (21–36.2)
	RISS-II	5.98 (5.28–7.2)	57.1 (52.5–61.5)	2.66 (2.4–2.86)	24.1 (20.1–28.3)
	RISS-III	2.58 (2.21–3.76)	31.6 (22.1–41.5)	1.31 (0.97–1.62)	3.8 (0.9–10.5)
	Total	6.29 (5.60–7.20)	58 (54.3–61.6)	2.5 (2.32–2.67)	22.5 (19.3–25.9)
New Score	0	9.01 (8.47–NA)	75.5 (69.4–80.6)	3.06 (2.64–3.33)	28.9 (23.0–35.1)
	1	5.98 (5.21–7.52)	57.9 (51.7–63.6)	2.69 (2.41–2.99)	26.3 (20.8–32.0)
	2	4.49 (3.50–5.48)	45.4 (36.8–53.7)	2.26 (2.02–2.61)	11.5 (6.1–18.6)
	3	1.93 (1.66–2.58)	22.5 (12.7–34.1)	1.18 (0.73–1.38)	5.0 (1.0–14.3)
	4	1.36 (0.34–NA)	30.8 (9.5–55.4)	0.79 (0.22–1.01)	0
	Total	6.29 (5.60–7.20)	58.0 (54.3–61.6)	2.5 (2.32–2.67)	22.5 (19.3–25.9)

Abbreviations: CI, confidence interval; ISS, International Staging System; NA, not applicable; OS, overall survival; PFS, progression-free survival; RISS, Revised International Staging System.

**Table 3:** OS and PFS analysis across three different diagnostic scores in relapsed/refractory patients (Cohort 2)

*Diagnostic Test*	*Cohort*	*Median OS years (95% CI)*	*2-year OS % (95% CI)*	*Median PFS years (95% CI)*	*2-year PFS % (95% CI)*
ISS	ISS-I	4.16 (3.40–4.92)	74.6 (67.6–80.4)	0.78 (0.62–0.92)	25.6 (19.7–31.8)
	ISS-II	2.09 (1.83–2.69)	50.8 (44.7–56.5)	0.49 (0.40–0.60)	15.9 (12.2–20.0)
	ISS-III	0.93 (0.65–1.22)	25.9 (18.7–33.6)	0.28 (0.23–0.36)	8.5 (4.7–13.7)
	Total	2.35 (1.93–2.93)	52.2 (48.1–56.2)	0.47 (0.41–0.56)	17.1 (14.3–20.0)
RISS	RISS-I	4.28 (3.32–NA)	83.5 (72.9–90.2)	1.06 (0.81–1.51)	27.4 (18.1–37.5)
	RISS-II	1.98 (1.75–2.64)	48.7 (41.4–55.5)	0.55 (0.45–0.64)	15.0 (10.8–19.9)
	RISS-III	0.83 (0.40–1.23)	18.5 (8.1–32.3)	0.25 (0.16–0.38)	9.6 (3.5–19.2)
	Total	2.37 (1.96–3.15)	52.2 (48.1–56.2)	0.60 (0.49–0.70)	17.0 (13.3–21.1)
New Score	0	4.01 (2.99–5.34)	76.2 (66.5–83.4)	0.89 (0.71–1.10)	24.7 (17.3–32.8)
	1	2.27 (1.79–4.54)	51.7 (41.3–61.2)	0.62 (0.48–0.79)	18.9 (12.4–26.5)

Abbreviations: CI, confidence interval; ISS, International Staging System; OS, overall survival; PFS, progression-free survival; RISS, Revised International Staging System.
