# Supplementary material for: Clinical utility of the Revised International Staging System in unselected patients with newly diagnosed and relapsed multiple myeloma
Source: Blood Cancer J. 2017 Feb 17;7(2):e528–. doi: 10.1038/bcj.2017.13 (PMC5386331; doi:10.1038/bcj.2017.13)
Supplement: Supplementary Table 1 [file bcj201713x1.docx]

**Supplementary Table 1: OS and PFS analysis across 3 different diagnostic scores in newly diagnosed patients (Cohort 1).**

| **Diagnostic Test** | **Cohort** | **Median OS years**  **(95% CI)** | **5 year OS % (95% CI)** | **Median PFS years (95% CI)** | **5 year PFS % (95% CI)** |
| --- | --- | --- | --- | --- | --- |
| **RISS** | **RISS-I** | **9.95**  **(8.62 – NA)** | **76.3**  **(68.8 – 82.3)** | **2.99**  **(2.54 – 3.44)** | **27.9**  **(20.6 - 35.7)** |
|  | **RISS-II** | **5.74**  **(5.23 – 6.42)** | **55.7**  **(51.7 – 59.5)** | **2.57**  **(2.36 – 2.75)** | **22.4**  **(19.0 - 26.0)** |
|  | **RISS-III** | **2.58**  **(2.21 – 3.20)** | **29.5**  **(21.8 – 37.5)** | **1.4**  **(1.22 – 1.66)** | **6.9**  **(3.2 -12.7)** |
|  | **TOTAL** | **5.67**  **(5.33 – 6.37)** | **55.4**  **(52.2 – 58.6)** | **2.44**  **(2.30 – 2.62)** | **21.3**  **(18.5 - 24.2)** |
|  | **Total** | **6.29**  **(5.60 – 7.20)** | **58.0**  **(54.3 - 61.6)** | **2.5**  **(2.32 – 2.67)** | **22.5**  **(19.3 - 25.9)** |
